# Diversity and temperature indirectly reduce CO_2_ concentrations in experimental freshwater communities

**DOI:** 10.1007/s00442-020-04593-0

**Published:** 2020-01-16

**Authors:** Leah Lewington-Pearce, Ben Parker, Anita Narwani, Jens M. Nielsen, Pavel Kratina

**Affiliations:** 1 School of Biological and Chemical Sciences, Queen Mary University of London, London, E1 4NS UK; 2 Department of Aquatic Ecology, Swiss Federal Institute of Aquatic Science and Technology, 8600 Dübendorf, Switzerland; 3 Present Address: National Oceanic and Atmospheric Administration, Alaska Fisheries Science Center, 7600 Sand Point Way NE, Seattle, WA 98115 USA

**Keywords:** Biodiversity, Climate warming, Consumer–resource interactions, Ecosystem functioning, Indirect effects, Trophic interactions

## Abstract

Biodiversity loss and climate warming are occurring in concert, with potentially profound impacts on ecosystem functioning. We currently know very little about the combined effects of these changes on the links between the community structure, dynamics and the resulting in situ CO_2_ concentrations in freshwater ecosystems. Here we aimed to determine both individual and combined effects of temperature and non-resource diversity (species inedible for a given consumer) on CO_2_ concentration. Our analysis further aimed to establish both direct effects on CO_2_ concentrations and potential indirect effects that occur via changes to the phytoplankton and zooplankton biomasses. Our results showed that there were no interactive effects of changes in temperature and diversity on CO_2_ concentration in the water. Instead, independent increases in either temperature or non-resource diversity resulted in a substantial reduction in CO_2_ concentrations, particularly at the highest non-resource diversity. The effects of non-resource diversity and warming on CO_2_ were indirect, resulting largely from the positive impacts on total biomass of primary producers. Our study is the first to experimentally partition the impacts of temperature and diversity on the consumer–resource dynamics and associated changes to CO_2_ concentrations. It provides new mechanistic insights into the role of diverse plankton communities for ecosystem functioning and their importance in regulating CO_2_ dynamics under ongoing climate warming.

**Electronic supplementary material:**

The online version of this article (10.1007/s00442-020-04593-0) contains supplementary material, which is available to authorized users.

## Introduction

Biodiversity loss and climate warming are both occurring simultaneously and at unprecedented rates (Butchart et al. 2010; IPCC 2014). Yet, very little is known about their combined effects on community structure, dynamics and on the flux of in situ CO_2_ between aquatic systems and the atmosphere (Atwood et al. 2015; Traill et al. 2010). A detailed assessment of the relationship between community structure and ecosystem function across combined diversity and temperature gradients is pivotal in understanding the role that biodiversity can play in mitigating the impact of climate warming.

Human activities have increased the concentrations of heat-trapping gases in the atmosphere, inducing climate warming at a rate that is still accelerating (Karl et al. 2015). Climate models forecast mean rises in global surface temperatures of 1.5 to 4.5 °C by the year 2100, with CO_2_ being the main contributor (IPCC 2014; Meinshausen et al. 2011). Freshwater communities are particularly sensitive to warming because they are often spatially confined, strongly size-structured and dominated by ectotherms, whose contributions to ecosystem functioning largely depend on environmental temperature (Sommer and Lewandowska 2011; Woodward et al. 2012). Ectotherms include diverse phytoplankton taxa that play a key role in carbon sequestration in freshwaters, through primary production (Davidson et al. 2015; Kratina et al. 2012; Low-Décarie et al. 2014). Climate warming can alter the ratio of heterotroph to autotroph plankton and shift the rates of photosynthesis and community respiration, two biological processes that drive the global carbon cycle and concentrations of atmospheric CO_2_ (Allen et al. 2005). Greater sensitivity of consumers to temperature, compared to producers, can amplify top-down control by increasing interaction strengths (Eklöf et al. 2015; Gaedke et al. 2010; Kratina et al. 2012; O'Connor et al. 2009). Increased herbivory can indirectly enhance emissions of CO_2_ into the atmosphere by reducing the phytoplankton biomass (Atwood et al. 2013). Alternatively, zooplankton grazers may be negatively affected by warming as their metabolic demands increase faster with raising temperatures then their grazing rates. (Fussmann et al. 2014; Rall et al. 2010). Consequently, rising temperatures could negatively influence body size and population growth rates of zooplankton, releasing phytoplankton from top-down control. Depending on the relative impacts of temperature on trophic interactions and community structure, warming may either reduce or increase CO_2_ concentrations in aquatic ecosystems, in addition to the general reduction of CO_2_ soluability at higher water temperates (Wiebe and Gaddy 1940).

Species diversity may alter the effects of warming on community structure, dynamics and CO_2_ concentration. For example, lakes with diverse phytoplankton communities contain many large species that are inedible to most herbivorous zooplankton that have limited gape or filtering apparatus (Hillebrand and Cardinale 2004). These ‘non-resource’ species are too large to be eaten by the zooplankton, but they may interfere with zooplankton ability to feed on their preferred phytoplankton resources (Hammill et al. 2015; Kratina et al. 2007; Narwani and Mazumder 2010). Such an increased ratio of non-resource to resource species in more diverse phytoplankton communities can reduce the strength of top-down control (Kratina et al. 2007; Narwani and Mazumder 2010), increase total phytoplankton biomass and consequently reduce concentration of CO_2_ in the water (Davidson et al. 2015). Similarly, diverse phytoplankton communities are more efficient in capturing essential resources and converting those into standing phytoplankton biomass than communities composed of fewer species, thus absorbing dissolved CO_2_ at a faster rate (Cardinale et al. 2011). However, we currently know very little about the combined effects of diversity and temperature on ecological community structure and dynamics, and particularly on the resulting changes in in situ CO_2_ concentrations. The carbon metabolism and carbon balance are inherently dynamic processes but it is unknown how closely CO_2_ concentrations track the dynamics of plankton communities.

Previous research commonly examined only the independent effects of diversity (Naeem et al. 1994; Schleuss et al. 2014) and temperature (Davidson and Janssens 2006) on carbon storage in terrestrial ecosystems, despite the fact that freshwater ecosystems emit a similar amount of CO_2_ due to changing land-use patterns (Cole et al. 2007). Few studies have investigated the combined effect of species richness and temperature on ecosystem processes in aquatic environments. Perkins et al. (2015) investigated the diversity of benthic macro-invertebrates and found that at low and high temperatures, multifunctionality increased with species richness so that approximately two species were required to drive an additional ecosystem process. However, it is still poorly understood how closely ecosystem function tracks community structure over time, including the specific roles of consumers, resources and non-resources on CO_2_ dynamics.

Therefore, we experimentally tested both the independent and interactive effects of temperature and gradients of non-resource diversity (different community structures) on CO_2_ concentrations. Corresponding time-series of consumer and resource densities, total phytoplankton biomass and CO_2_ concentrations were established for 96 experimental plankton communities. We hypothesized that higher temperature causes an indirect increase of CO_2_ concentration in the water by enhancing consumer respiration and intensifying consumer grazing on phytoplankton. We also partitioned the indirect effects of temperature on CO_2_ concentration due to the changes in plankton community structure from the direct effect of temperature due to the changes in solubility. By contrast, we expected that an increase of non-resource diversity indirectly reduce CO_2_ concentration by weakening consumer–resource interactions, increasing autotroph biomass and thus fixing of CO_2_ through photosynthesis. A greater freshwater carbon storage capacity can result from plant biomass being deposited in the sediment, thus escaping decomposition and re-mineralization in the water column. These hypothetically antagonistic impacts of temperature and diversity have the potential to further exacerbate or mitigate ongoing climate warming.

## Materials and methods

### Model communities and experimental design

We used the freshwater filter-feeding zooplankton *Daphnia pulex* (hereafter *‘D. pulex*’ or ‘consumer’) feeding on the freshwater green algae *Chlorella vulgaris* (hereafter ‘*C. vulgaris*’ or ‘resource’) as our consumer-resource model system. Daphnia are key zooplankton in many lentic ecosystems, with strong links to both phytoplankton resources and fish predators (Carpenter et al. 2001). The experimental design consisted of four phytoplankton diversity treatments consisting of 0 (only *C. vulgaris*), 2, 4 and 8 non-resource species, assembled into two different community compositions (A and B; Table 1) and of three different temperature treatments (19 °C, 23 °C and 27 °C). The two different community compositions allowed us to test the effect of diversity per se*,* as there may be variation in the composition of natural communities. The 14 species used in the non-resource phytoplankton species pool were selected for the following reasons (i) their cell (or colony) size was larger than ~ 45 μm and were, therefore, largely outside the dietary size spectrum for *D. pulex* (Narwani and Mazumder 2010); (ii) the species co-occur in natural lake ecosystems (US EPA’s National Lakes Assessment survey, Table 2); (iii) species could be distinguished morphologically under a microscope. Due to the limited size of the species pool, the two 8 non-resource species treatments inevitably shared some species with the 4 and 2 non-resource species compositions (Table 1).

**Table 1:** Experimental phytoplankton community compositions with individual species for each non-resource diversity treatment

No. of non-resource phytoplankton species	Phytoplankton species	
	Composition A	Composition B
2	*Closterium acerosum *	*Micrasterias crux-melitensis *
	*Cosmarium botrytis*	*Staurastrum pingue*
4	*Closterium acerosum *	*Closterium littorale *
	*Cosmarium botrytis *	*Eudorina elegans *
	*Micrasterias crux-melitensis *	*Micrasterias crux-melitensis *
	*Mougeotia* sp.	*Staurastrum pingue*
8	*Closterium acerosum*	*Ankistrodesmus falcatus*
	*Cosmarium botrytis *	*Closterium littorale *
	*Eremosphaera viridis *	*Cosmarium botrytis *
	*Eudorina elegans*	*Eremosphaera viridis *
	*Micrasterias crux-melitensis *	*Eudorina elegans *
	*Mougeotia* sp.	*Micrasterias crux-melitensis *
	*Pediastrum duplex *	*Staurastrum pingue *
	*Staurastrum pingue*	*Volvox aureus*

The 0 non-resource diversity treatment received only resource species *C. vulgaris*. All experimental assemblages received an equal amount of resource species *C. vulgaris* (1.6 × 10^6^ ESD) and had equivalent total biovolume of non-resource species (8 × 10^5^ ESD) in the 2, 4 and 8 diversity treatments

**Table 2:** Inocula of phytoplankton species that originated from the Experimental Phycology and Culture Collection of Algae at the University of Goettingen (EPSAG) and the Culture Collection of Algae of Charles University in Prague (CAUP)

Species	Source and Identifier	Cell Diameter (μm) (± SD) **Biovolume (μm**^**3**^**)**	No. lakes each species occurs
*Chlorella vulgaris*	EPSAG 211-11b	4 ± 0.44 **33.49**	403
*Cosmarium botrytis*	EPSAG 612-5	43 ± 1.6 **41608.66**	161
*Closterium acerosum*	EPSAG 126.8	333 ± 7.52 **109089**	545
*Mougeotia* sp.	EPSAG 650-1	1494 ± 67.06 **269181.69**	366
*Eremosphaera viridis*	EPSAG 228-1	182 ± 1.42 **3154950.59**	2
*Staurastrum pingue*	EPSAG 5.94	56 ± 3.3 **11084**	70
*Pediastrum duplex*	EPSAG 84.8	27 ± 6.52 **140**	360
*Eudorina elegans*	EPSAG 29.87	38 ± 2.83 **2467.7**	545
*Closterium littorale*	EPSAG 611-7	146 ± 7.74 **3750**	366
*Ankistrodesmus falcatus*	EPSAG 202-2	*44 ± 1.58* **363.2**	*2*
*Volvox aureus*	EPSAG 88-1	159 ± 13.34 **7563.1**	139
*Micrasterias crux-melitensis*	CAUP K602	98 +/98–1.13 **5670.6**	511

Cell diameter and biovolume estimates were determined from an average sample size of 30 cells calculated using a stage micrometer

Cell diameters are mean ± 1 SD. Biovolumes for each species were calculated using equations based on the body shape and cell size of each species (Hillebrand et al. 1999). The last column indicates the number of lakes each phytoplankton species occurs across North America as found in the US EPA’s National Lakes Assessment survey (2007)

### Inoculation biovolumes and experimental setup

Prior to the experiment, phytoplankton species were grown in batch monocultures in Bold’s Basal Medium (BBM) and the zooplankton were grown in batch culture with the green alga *Chlamydomonas reinhardtii* in sterile spring water (Volvic, France), which resembles the chemical composition of many freshwater ecosystems (see Table S1). Prior to inoculation, we measured the density and mean biovolume (estimated as the equivalent spherical diameter) of 30 natural units (cells, colonies or filaments) of each species monoculture used for the experiment, with a stage micrometer. The original sources of the phytoplankton taxa and their mean cell biovolumes at the time of inoculation can be found in Table 2. Species biovolumes were calculated using equations based on body shape and cell size of each phytoplankton species (Hillebrand et al. 1999). Experimental microcosms received a constant resource (*C. vulgaris*) biovolume of 1.6 × 10^6^ μm equivalent spherical diameter and a total biovolume of 8 × 10^5^ μm equivalent spherical diameter of all other appropriate species in the mixture, split equally between all non-resource species present (Tables 1, 2). This approach ensured that higher diversity treatments received the same total biovolume as the lower diversity treatments, regardless of different phytoplankton cell size. Each microcosm also received seven *D. pulex,* which were first acclimated to their assigned temperature treatment for three days prior to the experiment.

Experimental communities were reared in 1L glass media bottles filled with sterile mineral water that were haphazardly distributed within the incubators. We used commercial spring water for the experimental medium as preliminary tests using BBM resulted in extremely high phytoplankton densities and the rapid extinction of the zooplankton consumers. Media bottle tops were modified with small holes on the sides, large enough to prevent oxygen depletion and lethal build-up of toxic CO_2_ levels. However, the holes were small enough to prevent evaporative losses and minimize bacterial contamination. Side holes were only exposed when microcosms were inside the incubators, which had previously been sanitized with 70% ethanol.

All non-resource diversity treatments and species compositions were maintained at the three different temperatures in separate incubators (Stuart SI500, Orbital) set to 19 °C, 23 °C and 27 °C. Preliminary studies were used to determine the range of temperatures that enabled positive growth rates of all consumer, resource and non-resource species in monoculture. Incubators were lit with cool white LED light panels (Mirrorstone™) set to a 12 h light: 12 h dark cycle, to simulate natural diurnal changes in light. Each LED light panel emitted ca. 100 μmol m^−2^ s^−1^ of Photosynthetically Active Radiation (PAR). We collected 60 mL samples twice a week for 8 weeks, resulting in 16 temporal samples. The experiment consisted of two blocks (due to space limitation in the incubators) and all treatment combinations were replicated twice in each block, yielding a total of 96 experimental units (4 diversity treatments × 2 community compositions × 3 temperatures × 2 replicates × 2 blocks = 96).

### Sampling and sample processing

To homogenize the experimental communities and to ensure a representative sample, the microcosms were inverted and gently shaken, prior to each sampling, with bottle tops securely fastened and without the air holes exposed. All sampling and media replacement was done using sterile technique in a vertical lamina flow cabinet (PCR6, Labcaire), to prevent contamination. Each sample was microscopically inspected to ensure that there was no contamination of cultures with bacteria, fungi or protozoa over the duration of the study.

Each 60 mL sample was divided up into smaller sub-samples, to measure the CO_2_ concentration in the water, the density of consumer and resource and the total phytoplankton biomass. We estimated the phytoplankton biomass as chlorophyll-*a* concentration, because counting densities of all individual non-resource species over time was not logistically feasible. To measure CO_2_ concentration, the sample was transferred to 3 mL gastight vials (Labco), which were then sealed. Samples were taken during the light cycle to represent maximum CO_2_ uptake. A 500-μL headspace was introduced by withdrawing the sample and simultaneously replacing with 500 μL of oxygen free nitrogen via a needle and 3-way valve. After equilibration (30 min shaking), 100 μl samples were withdrawn from the headspace and injected into a gas chromatograph (GC) fitted with a flame-ionisation detector (Agilent Technologies; for details see Sanders et al. (2007). Headspace concentrations of CO_2_ were calculated from peak areas calibrated against known standards (Scientific and Technical Gases), and the total amount in the vial (headspace plus sample) was calculated using solubility coefficients (Weiss 1974; Yamamoto et al. 1976). Final CO_2_ concentrations were corrected for media addition days by subtracting the concentration of CO_2_ measured in control microcosms (only media without living organisms), measured at each experimental temperature treatment.

To estimate consumer density over time, two observers checked each experimental community for the presence of *D. pulex*. If *D. pulex* were present at low density, i.e. fewer than 20 individuals, we counted all the individuals in the microcosms (1L). If there were a greater number of individuals, we counted the number of individuals in the 60-mL sub-sample. To measure resource density (number of *C. vulgaris* cells), 10 mL sub-samples were fixed with Lugol’s iodine solution. *C. vulgaris* density was estimated by counting cells using a haemocytometer under a compound light microscope at 40 × magnification.

To estimate total phytoplankton biomass, we filtered 30 mL sub-sample onto glass fiber filters (Whatman, Grade 1, 25 mm) and stored them at  − 20 °C for chlorophyll-*a* measurements. We extracted the chlorophyll-*a* in acetone (90% *v/v* with ultra high purity water) for 24 h in a dark refrigerator. We used a spectrophotometer and measured absorption of light at 665 nm (Dalsgaard 2000). We replaced the volume sampled with 120 mL of sterile spring water starting from day 10, and continuing weekly. After each sampling event, bottles were placed back into incubators (with lids exposing air holes to allow gas exchange) in a haphazard fashion to eliminate edge effects.

### Statistical analyses

We analysed the independent and interactive effects of non-resource diversity and environmental temperature on four continuous response variables: (i) time-averaged consumer density (number of individuals L^−1^), (ii) time-averaged resource density (number of *C. vulgaris* cells per mL), (iii) time-averaged total phytoplankton biomass (aggregated biomass of all phytoplankton taxa in the community) and (iv) time-averaged concentration of CO_2_ (amount of CO_2_ in the water).

To assess the effects of the treatments and their interactions, we used linear mixed effects (LME) models with non-resource diversity and environmental temperature as fixed effects. We accounted for the temporal blocks, non-resource community composition and position of the microcosms in the incubators as random effects. We used the *varIdent* function to improve homogeneity of variance in the model fit (Zuur et al. 2009). This model represented a good fit to the data for all response variables, as denoted by the $R^{{2}}$ values (Nakagawa and Schielzeth 2013) (Table S2). Moreover, we fit this LME model to the time-series across the entire experiment and included time into the random factor term (Table S3). We also fit this same LME model to the time-series that accounted for temporal autocorrelation instead of time in the random factor term. Statistical outcomes of LMEs including all time-series data with and without temporal autocorrelation and time-averaged data were qualitatively identical; therefore we present the time-averaged model outputs only (Table S2). All analyses were performed in R 3.2.3 (R Core Team 2018) using the function *lme* in the package *nlme,* and *r.squaredGLMM* in the package *MuMIn*.

We then tested whether temperature had a direct effect on CO_2_ concentration due to the lower solubility of CO_2_ at higher temperatures (Wiebe and Gaddy 1940), or indirect effect due to the shift in community structure. To separate the physico-chemical effects from the biological effects of community structure, we adjusted the entire data set to the lowest temperature treatment (19 °C); therefore, CO_2_ concentrations measured at 23 °C were increased by 10.2% and CO_2_ concentrations measured at 27 °C were increased by 20.5%. These adjustments were based on the CO_2_ measurements in the control microcosms with no organisms, incubated at the three experimental temperatures. We then applied a piecewise structural equation modelling (SEM) approach (Lefcheck 2016) to both corrected and uncorrected data and tested whether the changes in CO_2_ concentrations resulted directly from the experimental diversity and temperature manipulations, indirectly through the changes in community structure, or from the effect of temperature on CO_2_ solubility (Atwood et al. 2015). The path diagrams (Fig. S1 supplementary material) expressed as a set of linear structured equations represented our biologically relevant hypotheses which were then evaluated individually. SEMs incorporated random effects of block, position in the incubator, non-resource composition and an additional temporal autocorrelation term for each day of the experiment. To test the directed separation of linear models, we used a Fisher’s C test following the piecewise SEM function as proposed by Lefcheck (2016). The Fisher’s C statistic was also used to obtain AIC values of the models. We compared seven different models and selected the model with the lowest AIC score, representing the best fit to our data (model 1, Table S4). The piecewise SEM returned parameter estimates and partial correlations, allowing our hypotheses to be tested at a significance level *α* = 0.05 (Fig. S1).

## Results

### Ecosystem function

**Fig. 1:**
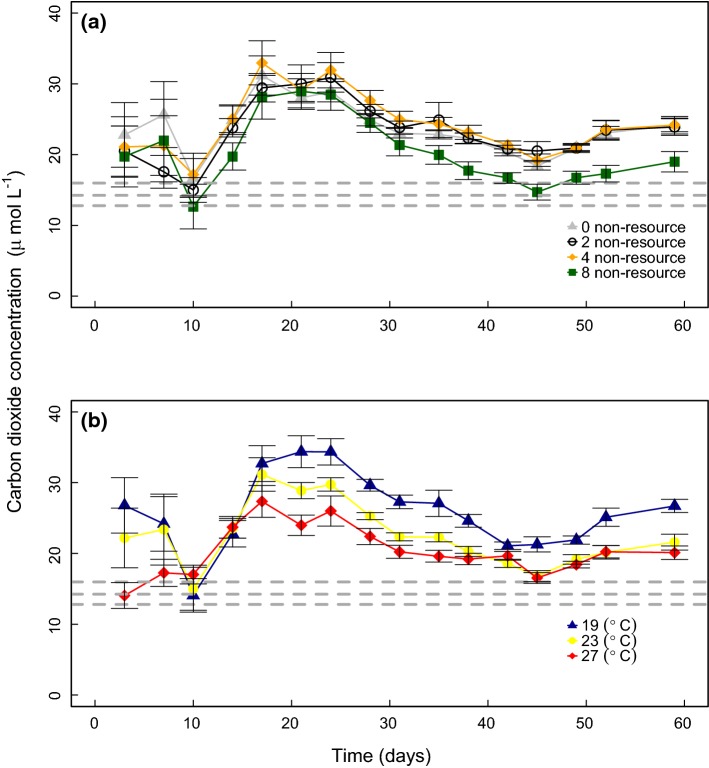
The independent effects of non-resource diversity (**a**) and environmental temperature (**b**) on the temporal dynamics of CO2 concentration. Points represent mean of *N* = 24 replicates ± 1 standard error for each diversity treatment level and mean of *N* = 32 replicates ± 1 standard error for each temperature treatment level. Dashed lines represent CO2 concentration at 19 °C, 23 °C and 27 °C, atmospheric equilibration is 15.98 μmol L-1, 14.24 μmol L-1 and 12.79 μmol L-1 respectively. **a** Higher non-resource diversity (LME, F_1,89_ = 9.719, *P* < 0.001) and **b** elevated temperature (LME, F_1,89_ = 48.942, *P* < 0.001) both independently reduced mean CO2 concentration in the water

Both increased non-resource diversity (LME, *P* < 0.0001; Table S3; Fig. 1a) and elevated temperature (*P* < 0.0001; Table S3; Fig. 1b) independently reduced mean CO_2_ concentration in the water. There was no interactive effect of temperature and diversity on mean CO_2_ concentration (*P* < 0.931; Table S3). The CO_2_ peak around day 20 (Fig. 1) coincided with an increase in consumer density and a decline in available resource (Fig. S2). After this peak observed in all treatment combinations, the highest non-resource diversity treatment diverged from the other treatments, with its CO_2_ concentration declining closer to the atmospheric equilibrium compared to lower non-resource diversity treatments (Fig. 1a). Since there were no qualitative differences in outcomes between time-series and time-averaged models that accounted for time, we focus the subsequent analyses on the time-averaged results.

**Fig. 2:**
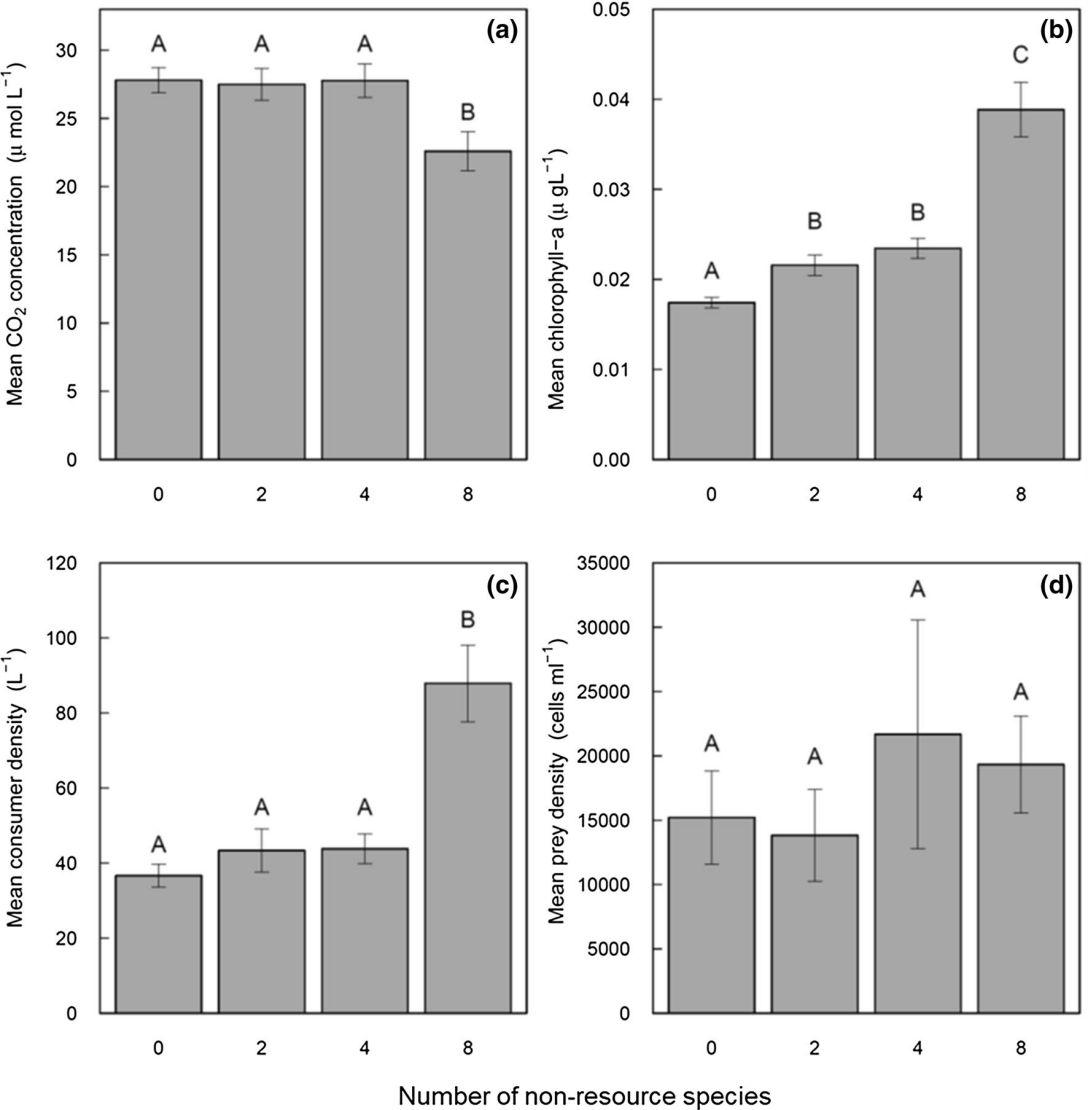
The effects of non-resource diversity on time-averaged CO_2_ concentration (**a**), time-averaged total phytoplankton biomass (**b**), time-averaged consumer density (**c**) and time-averaged resource density (**d**). Each bar represents means across all time points and temperature treatments (*N* = 24 replicates); error bars represent ± 1 standard error

**Fig. 3:**
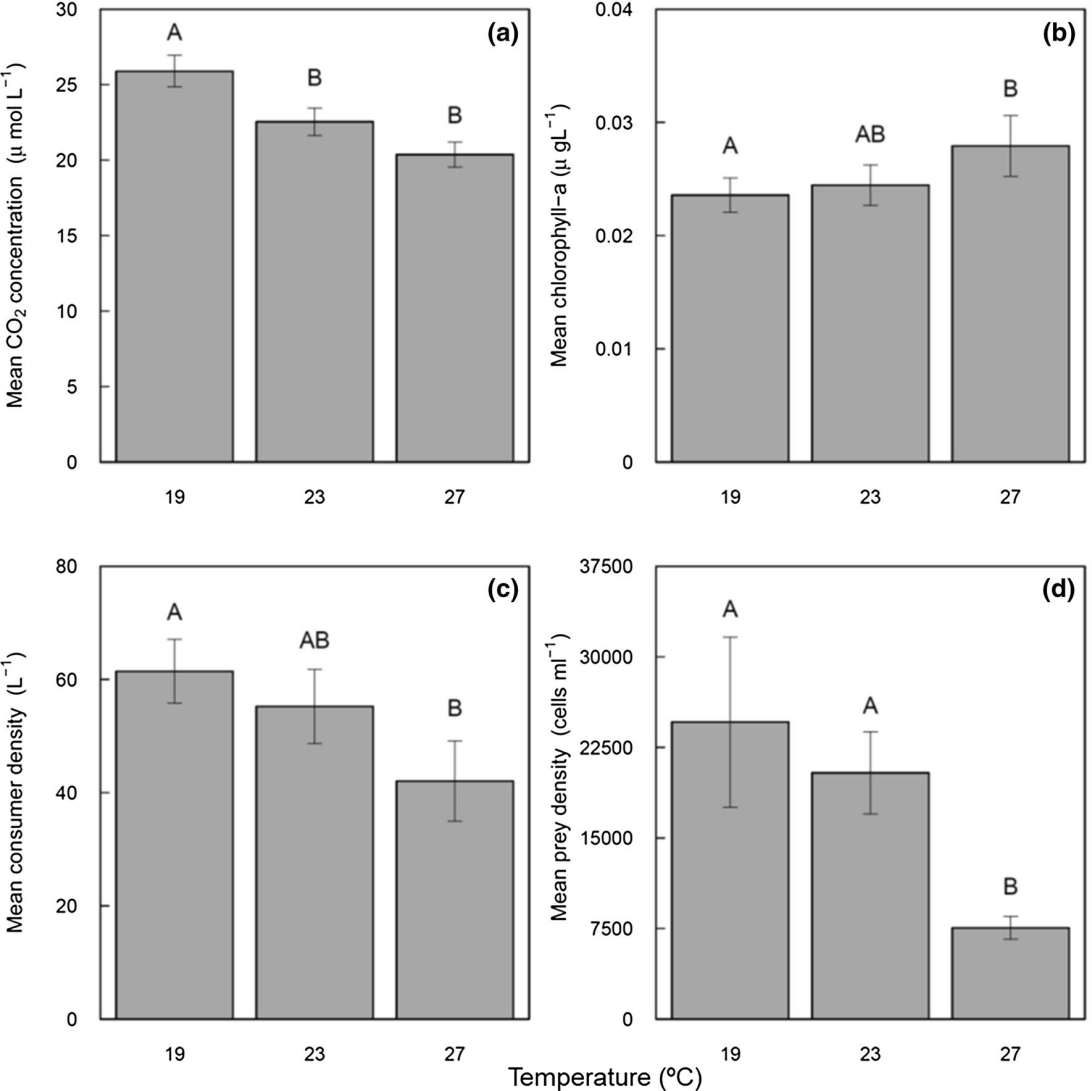
The effects of environmental temperature on time-averaged CO_2_ concentration (**a**), time-averaged total phytoplankton biomass (**b**), time-averaged consumer density (**c**) and time-averaged resource density (**d**). Each bar represents means across all time points and diversity treatments (*N* = 32 replicates); error bars represent ± 1 standard error

Higher non-resource diversity (LME, *F*_1,89_ = 9.719, *P* < 0.001; Fig. 2a, Table S2) and elevated temperature (LME, *F*_1,89_ = 48.942, *P* < 0.001; Fig. 3a, Table S2) both independently reduced time-averaged CO_2_ concentration in the water. There was no interactive effect of temperature and diversity on time-averaged CO_2_ concentration (LME, *F*_1,89_ = 0.838, *P* = 0.407; Table S2). There were significant differences in CO_2_ concentrations only between the highest (8 non-resource) diversity treatment and all other diversity treatments (Fig. 2a). The 8 non-resource diversity treatment reduced CO_2_ concentration by 15.4% compared to treatment with 0 non-resource (post-hoc Tukey’s HSD; 8–0 non-prey: *P* < 0.005; 8–2: *P* = 0.011; 8–4: *P* = 0.007). CO_2_ concentration declined by 14.9% when temperature was raised from 19 °C and 23 °C (post-hoc Tukey’s HSD; *P* = 0.022), by 27.1% when temperature was raised from 19 to 27 °C (post-hoc Tukey’s HSD; *P* < 0.001), but did not significantly change when temperature was raised from 23 to 27 °C (*P* = 0.324, Fig. 3a).

### Community structure

Alongside a reduction in time-averaged CO_2_ concentration, total phytoplankton biomass (LME, *F*_1,89_ = 60.931, *P* < 0.001; Fig. 2b, Table S2) and consumer density (LME, *F*_1,89_ = 13.333, *P* < 0.001; Fig. 2c, Table S2) increased significantly in the 8 non-resource species diversity treatment. However, resource density was not affected by non-resource diversity (LME, *F*_1,89_ = 1.309, *P* = 0.256; Fig. 2d, Table S2). Temperature had a positive effect on time-averaged total phytoplankton biomass (LME, *F*_1,89_ = 3.788, *P* = 0.050; Fig. 3b, Table S3), but a negative effect on both consumer density (LME, *F*_1,89_ = 20.358, *P* < 0.001; Fig. 3c, Table S2) and resource density (LME, *F*_1,89_ = 10.023, *P* = 0.002; Fig. 3d, Table S2).

### Direct and indirect effects of diversity and temperature

**Fig. 4:**
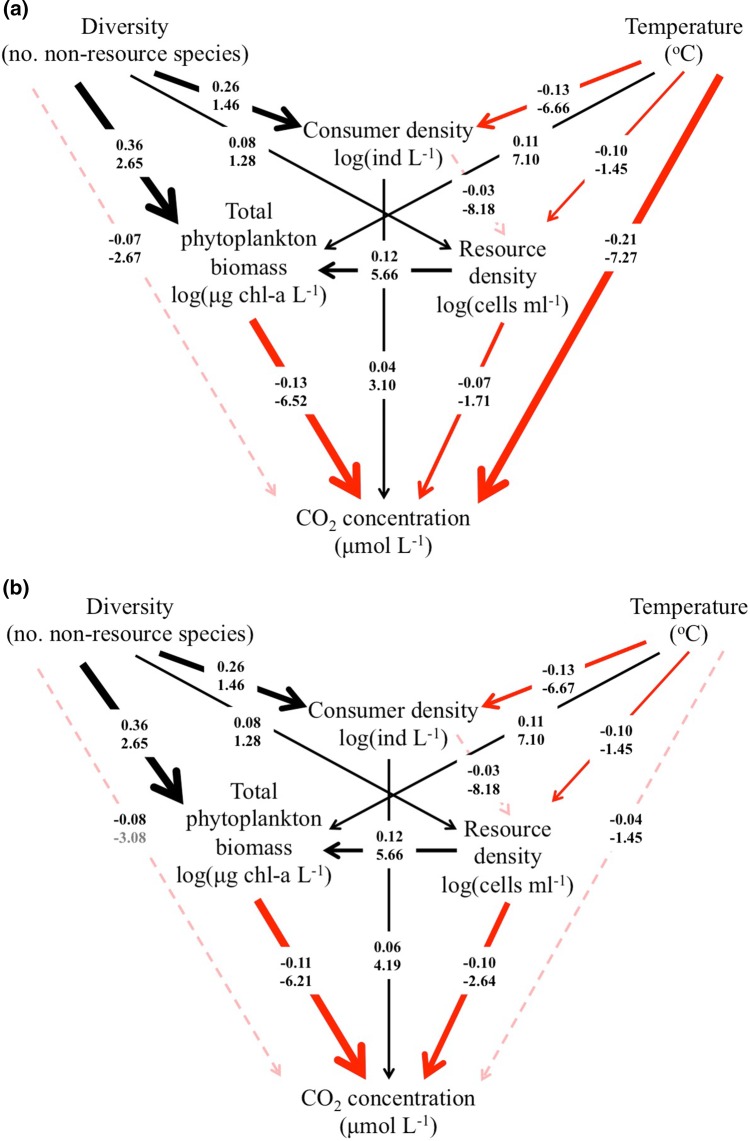
The best-fit structural-equation model (SEM) showing how the covariances among the variables predict the pathway of outcome of CO_2_ concentration. **a** Before correction for the effect of temperature on CO_2_ solubility in the water, the SEM retains a significant direct effect of temperature on CO_2_ concentration. **b** After correction for the effect of temperature on CO_2_ solubility, the SEM retains only an indirect effect of temperature on CO_2_ concentration. Significant direct pathways are displayed as solid lines (*P* < 0.05), while non-significant direct pathways are displayed as dashed lines. Red lines denote the negative effects; black lines denote the positive effects. The strength of the effect is proportional to the thickness of the lines and represented as the magnitude of the regression coefficients. Two types of path coefficients are placed next to corresponding pathways. Standardized regression coefficients (bold, black font) represent the standard deviation change in variable *Y* per unit change in variable *X*. Unstandardized regression coefficients (grey font) represent the standard deviation change in *Y,* given a standard deviation change in X. The amount of variation explained by the models was **(a)**$R^{{2}}$  = 0.30 for consumer density, R^2^ = 0.16 for total phytoplankton biomass, $R^{{2}}$ = 0.23 for CO_2_ concentration and $R^{{2}}$  = 0.02 for available resources; **(b)**$R^{{2}}$  = 0.30 for consumer density, $R^{{2}}$ = 0.16 for total phytoplankton biomass, $R^{{2}}$  = 0.26 for CO_2_ concentration and $R^{{2}}$ = 0.02 for available resources

Before accounting for reduced CO_2_ solubility at higher temperatures, the SEM showed a direct negative effect of temperature on CO_2_ concentration ( − 0.21, Fig. 4a), i.e. an increase of 1 × standard deviation (SD) in temperature resulted in a decrease of 0.21 SD in the concentration of CO_2_ in the water. However, after accounting for CO_2_ solubility, the direct effect of temperature disappeared and the SEM analyses instead supported that the combined indirect negative effects of temperature (standardized β =  − 0.04, *P* = 0.314) and diversity (standardized β =  − 0.08, *P* = 0.063) on CO_2_ concentration via shifts in the community structure (Fig. 4b). Diversity enhanced the total phytoplankton biomass (standardized β = 0.36, *P* < 0.001) and resource density (standardized β = 0.08, *P* = 0.045), indirectly reducing the CO_2_ concentration (standardized β = -0.11, *P* < 0.001 and standardized β =  − 0.10, *P* < 0.001, respectively). Furthermore, the indirect negative effect of diversity on CO_2_ via total phytoplankton biomass ( − 0.040, Fig. 4b) was stronger than the indirect negative effect of temperature ( − 0.012, Fig. 4b).

Density of consumers was positively affected by non-resource diversity (standardized β = 0.26, *P* < 0.001), resulting in increased CO_2_ concentrations (standardized β = 0.06, *P* = 0.021). The positive indirect effect of non-resource diversity on CO_2_ mediated by increased consumer density (0.016, Fig. 4b) was smaller than the negative effect on CO_2_ mediated through increased total phytoplankton biomass ( − 0.040, Fig. 4b). Temperature enhanced total phytoplankton biomass (standardized β = 0.11, *P* = 0.009) and reduced resource (standardized β =  − 0.10, *P* = 0.012) and consumer densities (standardized β =  − 0.13, *P* < 0.001), causing a net reduction in CO_2_ concentration. The negative effect of temperature on consumers was weaker than the positive effect of diversity, leading to a net positive effect of consumers on CO_2_ concentrations in the high-diversity and high-temperature treatments. There was no direct effect of consumer on resource density (standardized β =  − 0.03, *P* = 0.290).

## Discussion

We show that the responses of plankton communities to temperature and changing diversity can control the dynamics of CO_2_ concentrations in freshwater ecosystems. Our approach, combining gradients of experimental temperature and non-resource diversity, allowed us to tease apart their independent and interactive impacts. Increases in both temperature and non-resource diversity independently decreased CO_2_ concentrations in the water, with a substantial reduction in CO_2_ concentrations at the highest non-resource diversity. The effects of diversity and warming on CO_2_ were indirect, resulting largely from the positive impacts on total biomass of primary producers. The opposite diversity effect can be expected in ecosystems with high diversity of edible phytoplankton; however, more diverse resource assemblages often contain higher ratio of inedible to edible species for the focal consumers (Hillebrand and Cardinale 2004). We found no interactive effect of temperature and diversity on CO_2_ concentration, indicating that carbon capture by primary producers was the primary driver of CO_2_ dynamics.

Higher non-resource diversity indirectly reduced CO_2_ concentration in the experimental communities, through the positive effect on total phytoplankton biomass. However, the reduction of mean CO_2_ concentration was only visible at the highest non-resources diversity treatment with the highest total phytoplankton biomass. This suggests that non-resource diversity effects on some communities and ecosystem processes may only become evident after reaching a specific threshold. The existence of a diversity threshold has similarly been documented in studies manipulating consumer assemblages (Duffy et al. 2003) and indicates that experiments with a small number of species may overlook the effect of diversity on ecosystem functioning. There are at least three non-mutually exclusive mechanisms that could have increased consumer density at the highest non-resource diversity treatment: (i) non-resource species can facilitate resource species, (ii) a small proportion of inedible species can provide resource subsidy to the consumers and (iii) mean consumer body size was lower, allowing higher consumer density at the highest diversity treatment. However, the relative importance of these mechanisms remains to be tested. The CO_2_ concentrations at the highest diversity treatment were closer to atmospheric equilibration (Fig. 2a), but the experimental systems were still a net source of CO_2_, because Daphnia are efficient grazers on edible phytoplankton and also contribute CO_2_ via respiration. Our results correspond to the majority of freshwater lakes that are supersaturated with CO_2_ relative to the atmosphere, allowing a net flux of CO_2_ from the water column to the air by a concentration gradient (Cole and Caraco 1998; Cole et al. 2007).

In addition to the diversity effect per se, species identity of ecological communities is an important consideration when categorizing the impacts of diversity on ecosystem functioning (De Boeck et al. 2018). The composition of the non-resource community in the highest diversity treatment had different impacts because community composition B with 8 non-resource species had higher consumer density and phytoplankton biomass than composition A with 8 non-resource species (Fig. S3). Species identity can affect the long-term dynamics of edible (Behl and Stibor 2015) and inedible (Narwani and Mazumder 2012) phytoplankton and plays a major role in the net biodiversity effects on ecosystem functioning, contributing roughly 50% of the biodiversity effects across different ecosystems (Cardinale et al. 2011). Identifying species with traits that have key effects on ecosystem stability and functioning (De Boeck et al. 2018) is a fruitful avenue for future research. The photosynthetic responses of individual phytoplankton species differ in their sensitivity to temperature and to species interactions. In particular, warming can alter community dynamics through changes to the relative competitiveness of individual phytoplankton species (Lewington-Pearce et al. 2019). Some species benefit from increases in temperature and diversity if conditions favour their individual temperature optima (Huertas et al. 2011; Lewington-Pearce et al. 2019; Schabhüttl et al. 2013). Other taxa pushed away from their temperature optima can go locally extinct or experience competitive displacement from dominant species (Lewington-Pearce et al. 2019; Schabhüttl et al. 2013). This also stands true for the carbon capture abilities of individual phytoplankton species. In particular, cyanobacteria have very efficient carbon capture mechanisms, raising their internal concentration relative to their environment by a 1000-fold (Low-Décarie et al. 2014). In agreement with other studies (Pires et al. 2018b), our findings show that some species are more important than others in determining the community level response to biodiversity losses and climate warming.

CO_2_ concentrations were higher at 19 °C compared to 23 °C or 27 °C, suggesting the negative relationship between CO_2_ concentration and temperature. This contrasts with other work indicating increased CO_2_ emissions at higher temperatures (Allen et al. 2005; Lopez-Urrutia et al. 2006). The effect in our study is driven by lower CO_2_ solubility at higher temperatures (Wiebe and Gaddy 1940), but the direct effect of temperature on CO_2_ concentration was not retained in the best model after the data were corrected for solubility (Fig. 4b). Instead, the SEM analysis of corrected data revealed an indirect effect of temperature via an increase in phytoplankton biomass and a reduction in zooplankton density. This agrees with other studies indicating indirect effects of temperature on CO_2_ dynamics (Davidson et al. 2015).

There were no interactive effects of temperature and non-resource diversity on CO_2_ concentration. The independent negative effects of both non-resource diversity and warming on CO_2_ concentrations resulted from the increasing total phytoplankton community biomass. The SEM showed no significant relationship between phytoplankton biomass and consumers. This suggests that in our study, primary producers are the main drivers of the observed changes in CO_2_, by sequestration of carbon from the water into phytoplankton via photosynthesis (Watson et al. 1992). Consumers presumably altered CO_2_ concentration directly by respiratory losses and indirectly by reducing phytoplankton biomass. This highlights the importance of photosynthetic organisms in mitigating CO_2_ emissions into the atmosphere (Low-Decarie et al. 2011).

Although we analysed two different community compositions for each diversity level, a larger range of species and compositions can unequivocally tease apart the relative effects of diversity and species identity (Bell et al. 2009; Pires et al. 2018a). Five of the non-resource species in our study were shared between the two high-diversity compositions, precluding us from directly identifying non-resource species with the largest effect on the CO_2_ concentration or consumer dynamics. While not logistically feasible in our study, control monocultures and regular counts of all non-resource species would partition the expected additive effect of individual species from the observed effect of total phytoplankton biomass. As an example, larger phytoplankton species settle out of suspension faster than smaller species, which may have acted as a defence against grazing and contribute to losses of CO_2_ by organic carbon sedimentation (Tranvik et al. 2009). Our study also considered only a single zooplankton consumer. Although *Daphnia* spp. are keystone consumers in freshwater ecosystems (Carpenter et al. 2001), more diverse grazer communities consume a wider range of resources (Narwani and Mazumder 2010). Finally, enhancing the spatial extent of future studies is required to assess the effect of larger scale climatic changes on functioning of natural ecosystems (Pires et al. 2018a).

Mechanistic understanding of how climate warming and biodiversity loss impact the relationship between community structure and ecosystem function is a fundamental, yet still a largely unresolved aspect in ecology (Pires et al. 2018b). In natural systems, the impact of climate warming will be either weakened or exacerbated, depending on whether the temperature effect on phytoplankton richness is negative (Hillebrand et al. 2012; Petchey et al. 1999), positive (Yvon-Durocher et al. 2015) or neutral (Hillebrand et al. 2010; Kratina et al. 2012). Controlled microcosm experiments have been identified as an important tool to fill the gaps in the current understanding of how multiple stressors impact CO_2_ dynamics in freshwater systems (Hasler et al. 2016). Our experimental design allowed us to partition the effects of temperature and diversity and suggested that systems with more diverse non-resource communities may mitigate the pace of climate warming by increasing primary production and carbon capture and reducing the return of CO_2_ to the atmosphere by primary consumers. With this information in hand, we may begin to develop models that more realistically predict the impacts of changing biodiversity and climate warming on ecosystems.

## Electronic supplementary material

Below is the link to the electronic supplementary material.

Supplementary file1 (DOCX 9335 kb)
