# Supplementary material for: Diversity and temperature indirectly reduce CO2 concentrations in experimental freshwater communities
Source: Oecologia. 2020 Jan 16;192(2):515–27. doi: 10.1007/s00442-020-04593-0 (PMC7002461; doi:10.1007/s00442-020-04593-0)
Supplement: Supplementary file 1 — Supplementary file1 (DOCX 9335 kb) [file 442_2020_4593_MOESM1_ESM.docx]

**Supplementary Material:**

**Diversity and temperature indirectly reduce CO_2_ concentrations in experimental freshwater communities**

***Leah Lewington-Pearce ^1^, Ben Parker ^1^, Anita Narwani ^2^, Jens M. Nielsen^1,+^ & Pavel Kratina ^1^***

^1^School of Biological and Chemical Sciences, Queen Mary University of London, London E1 4NS, United Kingdom.

^2^Department of Aquatic Ecology, Swiss Federal Institute of Aquatic Science and Technology, Dübendorf 8600, Switzerland.

***^+^***Present address: National Oceanic and Atmospheric Administration, Alaska Fisheries Science Center, 7600 Sand Point Way NE, Seattle, WA 98115, USA


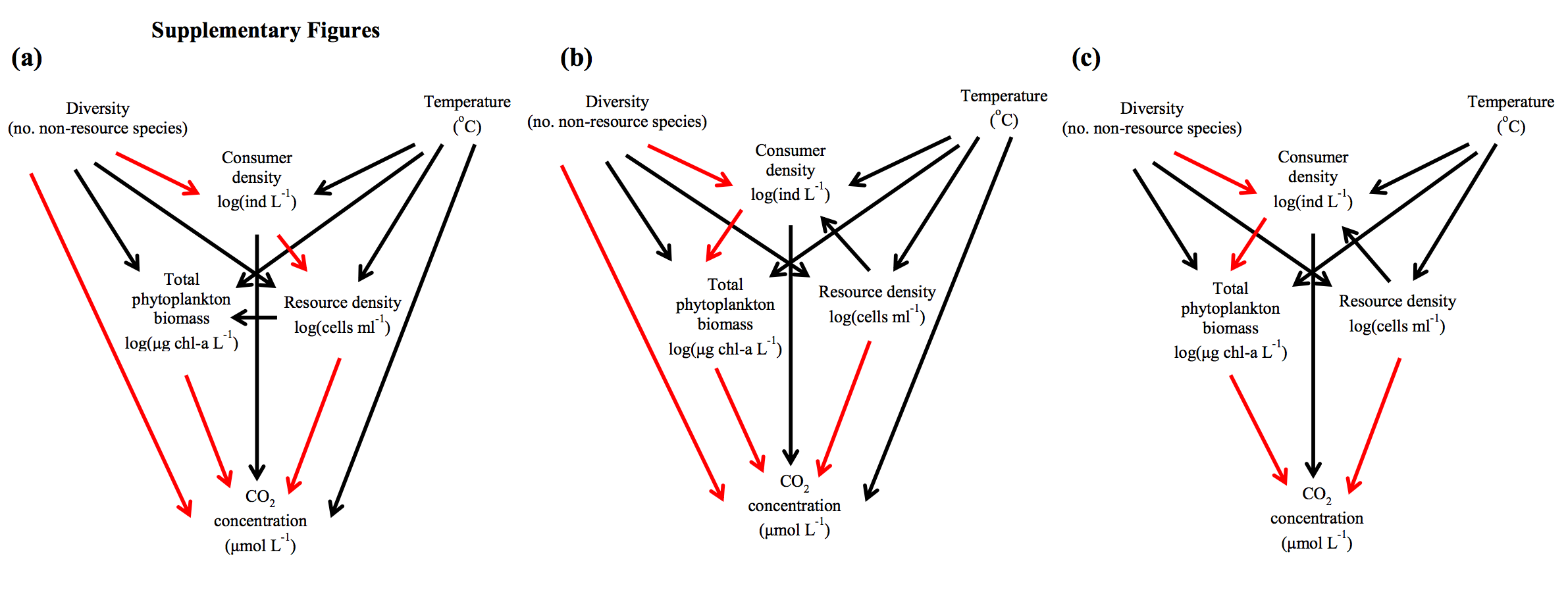


**Figure S1.** Conceptual path diagram illustrating the alternative hypotheses based on the models presented in Table S2.5, of how warming (^o^C) and phytoplankton diversity (number of non-resource species), may affect consumer density, resource density, total phytoplankton biomass (total chlorophyll-a concentration) and CO_2_ concentration. Red and black colors indicate negative and positive effects respectively. All models, (a) model 1 and 2, (b) model 3, (c) Model 4, reflect our hypotheses that (*i*) high temperature would directly increase consumer density, resource density and total phytoplankton biomass; (*ii*) high diversity would reduce consumer density, increase resource density and total phytoplankton biomass; (*iii*) consumers increase CO_2_ concentration whilst total phytoplankton biomass and resource density reduces CO_2_ concentration in the water. (*iv*) at high temperature, high diversity would reduce consumer density, increase resource density and total phytoplankton biomass, and reduce CO_2_ concentration; (*v*) at high temperature, low diversity would increase consumer density, decrease resource density, decrease total phytoplankton biomass but increase CO_2_ concentration. We composed models with (model 1 2 and 3) or without (model 4) a direct effect of temperature and diversity on CO_2_ concentration, and with biologically relevant combinations of direct links of consumer, resource densities and total phytoplankton biomass. Model 2 included phytoplankton composition as a random factor.

**
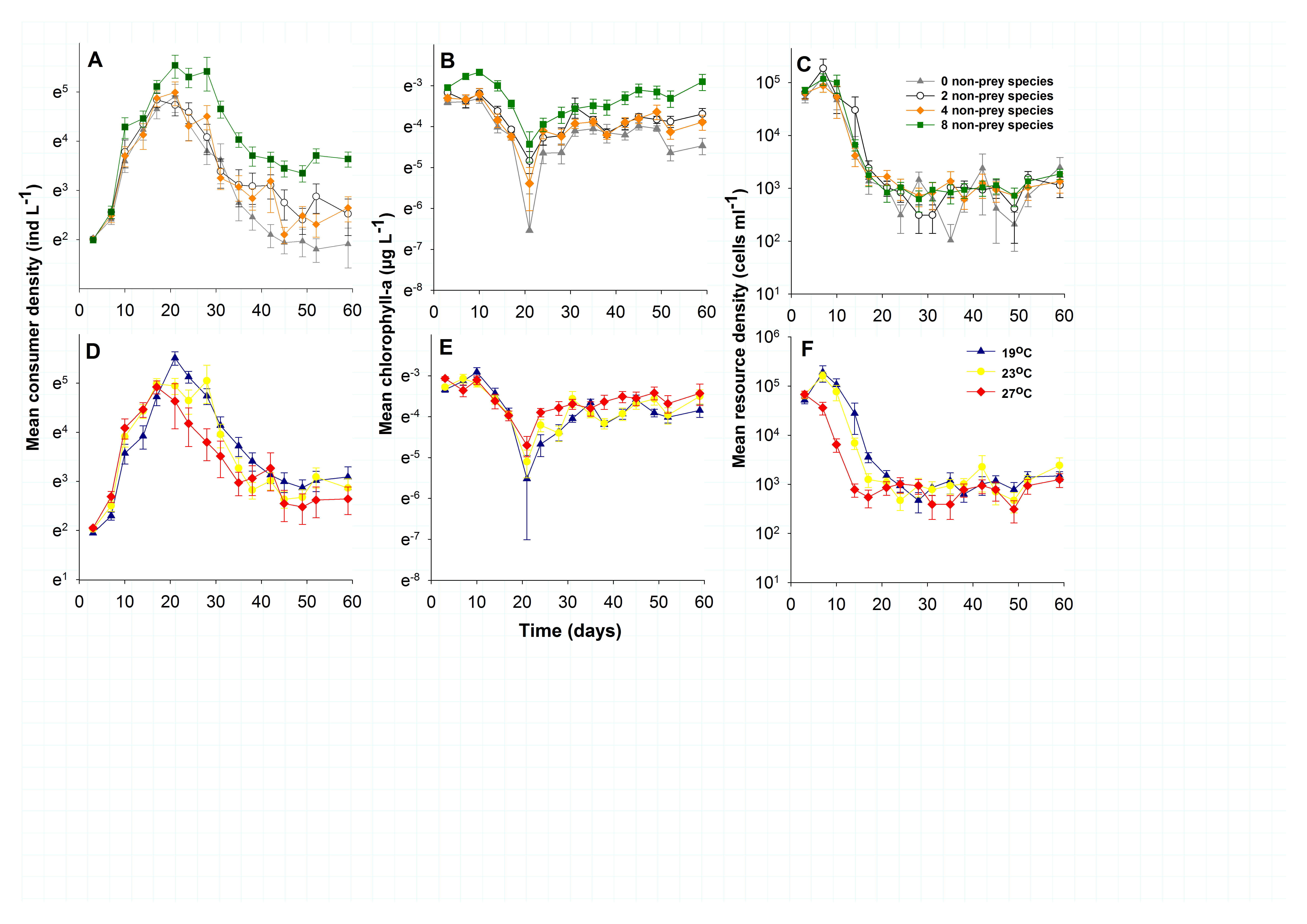
**

**Figure S2.** The effect of non-resource diversity (A-C) and temperature (D-F) on the community dynamics of consumer density (A and D), total phytoplankton biomass/chlorophyll-a (B and E) and resource density (C and F). Points represent mean of 24 replicates ± 1 standard error for each diversity treatment and 32 replicates ± 1 standard error for each temperature treatment.

**
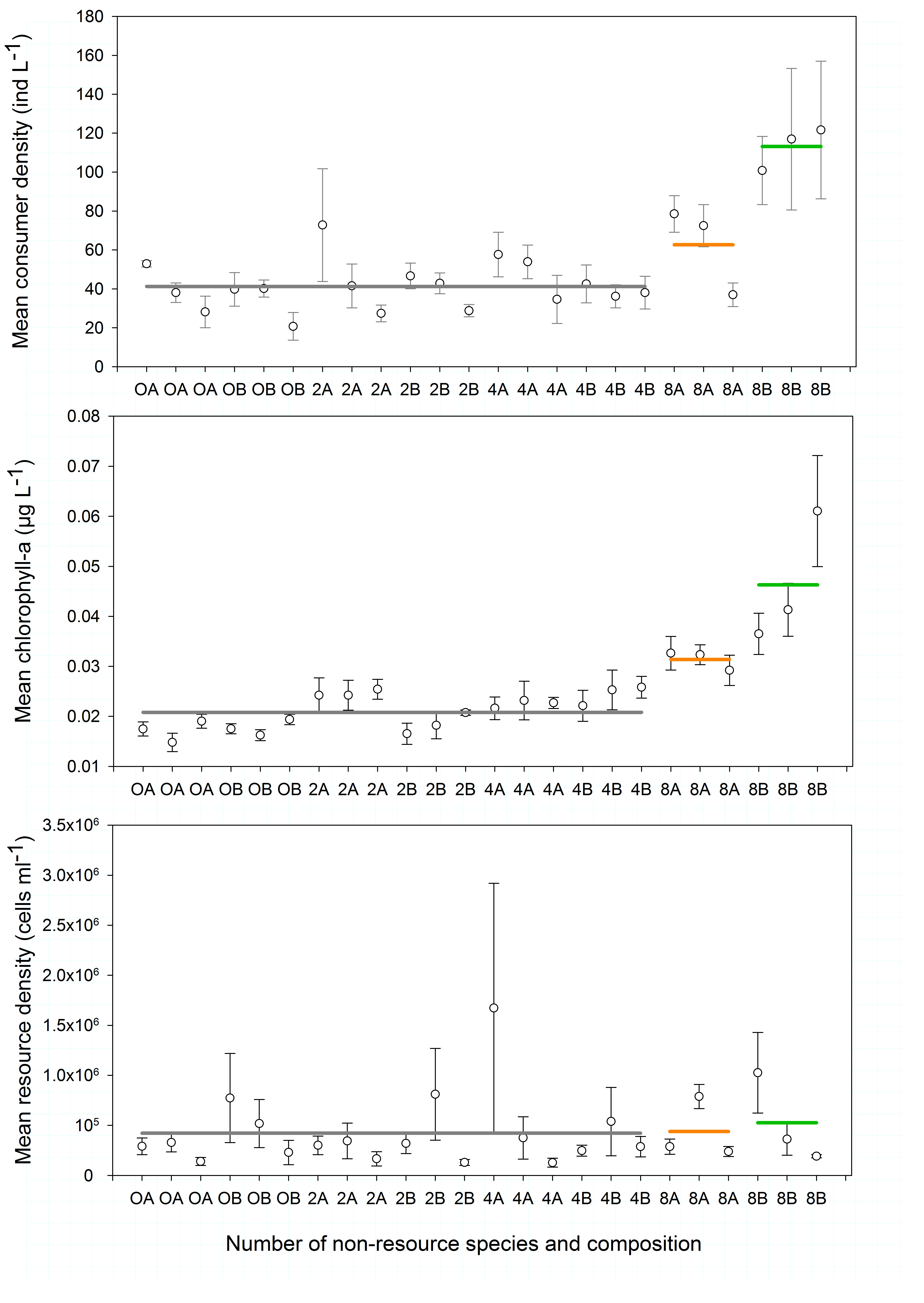
**

**Figure S3.** Mean consumer density, chorophyll-*a* concentration (total phytoplankton biomass) and resource density over the over the experimental duration for different diversity levels and community compositions (error bars are ± 1 standard error, N=4). Horizontal lines are means of each diversity category (grey for all 0, 2 and 4 non-resource diversity treatments combined, orange for 8 non-resource composition A and green for 8 non- resource composition B).

**Table S1.** Chemical composition of Volvic Mineral Water used as experimental media.

| Mineral: | Composition  (mg l¯¹): |
| --- | --- |
| Calcium (Ca) | 12 |
| Sulphates (SO₄) | 9 |
| Magnesium (Mg) | 8 |
| Sodium (Na) | 12 |
| Bicarbonates (HCO₃) | 74 |
| Potassium (K) | 6 |
| Silica (SiO₂) | 32 |
| Chlorides (Cl¯) | 15 |
| Nitrates (NO₃) | 7.3 |

**Table S2.** Linear mixed effects (LMEs) model summary statistics illustrating the independent and combined effects of non-resource diversity and environmental temperature on the time averaged response variables: (*i*) consumer density (number of *D. pulex* per sample), (*ii*) resource density (number of *C. vulgaris* cells per sample), (*iii*) total phytoplankton biomass (aggregated biomass of all phytoplankton taxa in the community) and (*iv*) concentration of CO_2_ (amount of CO_2_ in the water uncorrected for the difference in solubility at each temperature). Temperature and non-resource diversity were treated as fixed effects. We accounted for the temporal blocks, non-resource community composition and position of the microcosms in the incubators (nested in time) as random effects. We used the *varIdent* function to improve homogeneity of variance in the model fit (Zuur *et al.* 2009).

|  | d.f. | *F* value | R^2^ (cond) | R^2^ (marg) | *P* |
| --- | --- | --- | --- | --- | --- |
| (a) CO_2_ uncorrected |  |  | 0.938 | 0.565 |  |
| Diversity | 1,89 | 9.719 |  |  | 0.0025 |
| Temperature | 1,89 | 48.942 |  |  | <0.0001 |
| Diversity*Temperature | 1,89 | 0.042 |  |  | 0.838 |
|  |  |  |  |  |  |
| (b) Phytoplankton biomass |  |  | 0.609 | 0.236 |  |
| Diversity | 1,89 | 60.931 |  |  | <0.0001 |
| Temperature | 1,89 | 3.788 |  |  | 0.050 |
| Diversity*Temperature | 1,89 | 0.732 |  |  | 0.395 |
|  |  |  |  |  |  |
| (c) Consumer |  |  | 0.414 | 0.219 |  |
| Diversity | 1,89 | 13.333 |  |  | 0.0004 |
| Temperature | 1,89 | 20.358 |  |  | <0.0001 |
| Diversity*Temperature | 1,89 | 0.462 |  |  | 0.498 |
|  |  |  |  |  |  |
| (d) Resource |  |  | 0.638 | 0.099 |  |
| Diversity | 1,89 | 1.309 |  |  | 0.256 |
| Temperature | 1,89 | 10.023 |  |  | 0.002 |
| Diversity*Temperature | 1,89 | 0.353 |  |  | 0.554 |

**Table S3.** Linear mixed effects (LMEs) model summary statistics illustrating the independent and combined effects of non-resource diversity and environmental temperature on four response variables: (*i*) consumer density (number of *D. pulex* per sample), (*ii*) resource density (number of *C. vulgaris* cells per sample), (*iii*) total phytoplankton biomass (aggregated biomass of all phytoplankton taxa in the community) and (*iv*) concentration of CO_2_ (amount of CO_2_ in the water uncorrected for the difference in solubility at each temperature). This model was fit to the whole time series data. Temperature and non-resource diversity were treated as fixed effects. We accounted for the temporal blocks, non-resource community composition and position of the microcosms in the incubators (nested in time) as random effects. We used the *varIdent* function to improve homogeneity of variance in the model fit (Zuur *et al.* 2009).

|  | d.f. | *F* value | R^2^ (cond) | R^2^ (marg) | *P* |
| --- | --- | --- | --- | --- | --- |
| (a) CO_2_ uncorrected |  |  | 0.855 | 0.052 |  |
| Diversity | 1,191 | 18.161 |  |  | <0.0001 |
| Temperature | 1,1308 | 119.005 |  |  | <0.0001 |
| Diversity*Temperature | 1,1308 | 0.008 |  |  | 0.931 |
|  |  |  |  |  |  |
| (b) Phytoplankton biomass |  |  | 0.899 | 0.174 |  |
| Diversity | 1,191 | 125.591 |  |  | <0.0001 |
| Temperature | 1,1310 | 9.995 |  |  | 0.002 |
| Diversity*Temperature | 1,1310 | 3.686 |  |  | 0.055 |
|  |  |  |  |  |  |
| (c) Consumer |  |  | 0.799 | 0.046 |  |
| Diversity | 1,191 | 29.691 |  |  | <0.0001 |
| Temperature | 1,1310 | 28.164 |  |  | <0.0001 |
| Diversity*Temperature | 1,1310 | 0.976 |  |  | 0.323 |
|  |  |  |  |  |  |
| (d) Resource |  |  | 0.858 | 0.008 |  |
| Diversity | 1,191 | 1.911 |  |  | 0.169 |
| Temperature | 1,1310 | 13.612 |  |  | 0.0002 |
| Diversity*Temperature | 1,1310 | 0.627 |  |  | 0.429 |

**Table S4.** A comparison of different structural equations models (SEMs) used to explain patterns of covariance among variables analyzed in this experiment (Lefcheck 2016). We compared the models with (models 1,2 and 3) or without (model 4) a direct effect of temperature (T) and diversity (D) on CO_2_ concentration, and with biologically relevant combinations of direct links of consumer (C), resource (R) densities and total phytoplankton biomass (Chl). The best model (model 1) was also compared to a model with phytoplankton composition as a random factor (model 2) to test whether species compositions contributed to any variation in the data. We also simplified the model by removing individual pathway predictors (models 5 and 6) but model 1 described the data best, as indicated by the lowest AIC and non-significant Fisher C. For each model we report the degrees of freedom, Fisher C, P value, *n* (number of samples) and *K* (number of model parameters). We selected the model with the lowest AIC score, representing the best fit to our data. All SEMs incorporated a random effect of block, time and position within the incubator. The degrees of freedom were extracted directly from the analyses summary statistics using the *sem.fit* function from the PiecewiseSEM package in R. Notes: The best model is in bold font.

| **Model** | **Description** | **d.f.** | **Fisher C** | ***P*** | ***n*** | ***K*** | **AIC** |
| --- | --- | --- | --- | --- | --- | --- | --- |
| **1** | **CO_2_ ~ T + D + Chl + C + R**  **Chl ~ T + D + R**  **R ~ T + D + C**  **C ~ T + D** | **2** | **3.20** | **0.202** | **1536** | **33** | **69.2** |
| 2 | Model 1 with composition as random factor | 2 | 3.38 | 0.184 | 1536 | 37 | 77.38 |
| 3 | CO_2_ ~ T + D + Chl + C + R  Chl ~ T + D + C  R ~ T + D  C ~ T + D + R | 2 | 31.19 | 0 | 1536 | 33 | 97.19 |
| 4 | CO_2_ ~ Chl + C + R  Chl ~ T + D + C  R ~ T + D  C ~ T + D + R | 6 | 39.06 | 0 | 1536 | 31 | 101.06 |
| 5 | CO_2_ ~ T + D + Chl + C + R  Chl ~ T + D + R  R ~ T + C  C ~ T + D | 4 | 9.40 | 0.052 | 1536 | 32 | 73.4 |
| 6 | CO_2_ ~ T + D + Chl + C + R  Chl ~ T + D + R  R ~ T  C ~ T + D +R | 4 | 8.66 | 0.070 | 1536 | 32 | 72.66 |
